# Environmental and occupational respiratory diseases – 1032. Study of allergen pattern in cases of chronic allergic rhinitis in central India

**DOI:** 10.1186/1939-4551-6-S1-P31

**Published:** 2013-04-23

**Authors:** Subir Jain

**Affiliations:** 1 Ent Centre, India

## Background

To know the Allergen pattern of Central India in cases of Chronic Allergic Rhinitis. From year 2006 to 2012.

## Methods

504 patients of Chronic Allergic Rhinitis in the age group of 06 to 63 years of age of either sex were taken for study of Allergen Pattern by Modified Prick Test.

After detailed history. Clinical Examination of ENT, Routine blood counts, absolute Eosionophil count, Serum IgE estimation. Patient were off the antihistaminic for minimum of seven days. Allergy test by modified prick method was performed on upper limbs on palmer aspect of forearms. Glycerinated Histamine acid phosphate as positive control and Glycerinated buffer saline as negative control were used. Each patient was tested for same 166 Allergens. Wheal & flare response recorded.

## Results

In above study with 504 patients following were the percentage of different Allergens positive in above group.

House dust mite 18% with D. Farinae 18%, Pollens 78.5% with Prosopis Juliflora 15.5% , Fungus 13.5% with Aspergillus Flavus 4.5%, Insects 64.5% with Cockroach female 35.5%, Dusts 38.5% with Grain Dust Rice 22% , Danders 21% with Human Dander 6.5%, Fabrics 5% with Silk 2%, Foods 50% with Milk 5%, Miscellaneous 7.5% with Parthenium Leaves 4%.

## Conclusions

The above study gave us the common Allergen pattern in Central part of India in cases of Chronic Allergic Rhinitis as Pollens 78.5%, Insects 64.5%, Food 50%, Dusts 38.5% were mostly responsible for Chronic Allergic Rhinitis.

